# The impact of government pandemic policies on the vulnerability of healthcare workers to SARS-CoV-2 infection and mortality in Jakarta Province, Indonesia

**DOI:** 10.1080/07853890.2023.2293306

**Published:** 2024-01-11

**Authors:** Rina Agustina, Davrina Rianda, Aly Lamuri, Karina Rahmadia Ekawidyani, Deviana Ayushinta Sani Siregar, Dyana Santika Sari, Prashti Mutia Wulan, Natasha Dianasari Devana, Ari Fahrial Syam, Ahmad Jabir Rahyussalim, Dwi Oktavia Handayani, Widyastuti Widyastuti, Anuraj Harish Shankar, Ngabila Salama

**Affiliations:** a Department of Nutrition, Faculty of Medicine, Universitas Indonesia – Dr. Cipto Mangunkusumo General Hospital, Jakarta, Indonesia; b Human Nutrition Research Center, Indonesian Medical Education and Research Institute (HNRC-IMERI), Faculty of Medicine, Universitas Indonesia, Jakarta, Indonesia; c Department of Nutrition, University of California at Davis, Davis, CA, USA; d Big Data Center, Indonesian Medical Education and Research Institute (BDC-IMERI), Faculty of Medicine, Universitas Indonesia, Jakarta, Indonesia; e Department of Community Nutrition, Faculty of Human Ecology, IPB University, Bogor, Indonesia; f Department of Internal Medicine, Faculty of Medicine, Universitas Indonesia – Dr. Cipto Mangunkusumo General Hospital, Jakarta, Indonesia; g Department of Orthopaedic and Traumatology, Faculty of Medicine, Universitas Indonesia – Dr. Cipto Mangunkusumo General Hospital, Jakarta, Indonesia; h Health Office, Government of Jakarta Province, Jakarta, Indonesia; i Oxford University Clinical Research Unit – Indonesia, Jakarta, Indonesia; j Centre for Tropical Medicine and Global Health, Nuffield Department of Medicine, University of Oxford, Oxford, UK

**Keywords:** COVID-19, healthcare worker, Indonesia, policy, SARS-CoV-2

## Abstract

**Introduction:**

Healthcare workers (HCWs) are on the frontlines of the COVID-19 pandemic, putting them at a higher risk of infection and disease than non-HCWs. We analysed the effects of government policies for the public and for HCWs on the likelihood of Severe Acute Respiratory Syndrome Coronavirus 2 (SARS-CoV-2) infection and mortality among HCWs during the first 8 months of the pandemic in Jakarta province, the capital city and COVID-19 hotspot in Indonesia.

**Methods:**

We conducted a retrospective cohort study using secondary data from the Jakarta provincial government from March to October 2020, which included sociodemographic characteristics, symptoms, comorbidities and COVID-19 diagnosis history for all cases. A generalized linear mixed-effect regression model was used to determine the effect of each month on the odds ratio (OR) of COVID-19 cases and deaths for HCW compared with non-HCW, assuming that monthly trends were influenced by varying government policies.

**Results:**

A total of 894,487 suspected and confirmed COVID-19 cases in health facilities in Jakarta province were analysed. The OR of confirmed cases for HCW was 2.04 (95% CI 2.00–2.08; *p* < .001) compared to non-HCW. Despite this higher OR for infection, the case fatality rate (2.32 per 100) and OR (1.02, 95% CI 0.93–1.11; p = .65) of COVID-19 deaths for HCW were similar to those of non-HCW. We observed a trend towards a lower number of COVID-19 patients in hospitals and lower odds of COVID-19 cases among HCWs during the April-to-July 2020 phase compared to the August-to-October phase. This chronologically aligned with more extensive policies to support hospital-based, community-based and well-being-related actions to protect HCW.

**Conclusions:**

HCW had higher odds of having SARS-CoV-2 infection, yet similar odds of death from COVID-19, as compared to non-HCW. Government policies with collective efforts to prevent hospital overcapacity during high transmission and burden periods of the pandemic should be prioritized.

## KEY MESSAGES

- Healthcare workers (HCWs) had higher exposure and odds of Severe Acute Respiratory Syndrome Coronavirus 2 (SARS-CoV-2) infection than non-HCWs but a similar risk of death, consistent with previous studies.
- Government policies favouring reduced workloads of HCW and interventions to promote resilience can be achieved through combined hospital-based, community-based and well-being-related approaches.
- Studies to identify the patterns and trends of COVID-19 cases and deaths, hospital admissions and policy dynamics are important to promote evidence-based decision-making by the government.

## Introduction

The mortality from Coronavirus Disease-2019 (COVID-19) had exceeded 6 million persons by December 2022, with a 2% global case fatality rate (CFR) [1]. Ongoing emergence of various mutations of Severe Acute Respiratory Syndrome Coronavirus 2 (SARS-CoV-2) has influenced infection and transmission, leading to repeated pandemic waves. SARS-CoV-2 sequences deposited to the Global Initiative on Sharing All Influenza Data (GISAID) from the Indonesian Consortium of SARS-CoV-2 Genomic Surveillance indicate variants circulating during the first pandemic wave were Alpha, Beta and the local variants B.1.466.2, B.1.1.398, B.1.470 and B.1.459 [2], followed later by Delta and Omicron variants. As seen in other countries, healthcare workers (HCWs) in Indonesia have been on the frontlines and their mortality from COVID-19 has been higher than for any other infectious disease outbreak in the history of the country [3,4]. Until mid-August 2021, there were 1891 HCW deaths due to COVID-19, and a surge of HCW deaths occurred during the Delta wave in mid-2021, wherein 114 doctors died despite being fully vaccinated [5,6]. This highlights the need to identify factors affecting HCW risk of infection and death, and how these change following public health measures and government policies [7].

There are multiple reasons HCWs are at a higher risk of COVID-19. First, as care providers the exposure to COVID-19 cases is high [7–9], which is exacerbated during periods of overcapacity of facilities [10,11]. In addition, HCW frequently work overtime as coworkers may suffer from COVID-19, thereby exposing remaining staff to even higher risk of infection [7,8,12]. Mahendradhata et al. found that the number of medical staff in Indonesia were insufficient to deal with the increased demand to manage COVID-19 cases [13]. In response, the Ministry of Health recruited health volunteers to enhance the workforce and set up a triage wherein COVID-19 suspect patients were sent to designated referral hospitals or isolation wards until proven otherwise [14,15]. Second, because comorbid conditions such as hypertension, diabetes mellitus and pneumonia also increase the risk of mortality, guidelines and practical approaches for HCW with comorbidities were created to reduce their risk of infection [9].

Institutional and governmental policies and support are necessary to reduce the risk for COVID-19 in HCW [16–18]. However, to date, only a few studies have been conducted to compare the likelihood of SARS-CoV-2 infection and mortality in HCW and non-HCW in response to government policies, particularly in low- and middle-income countries (LMICs), including Indonesia. Such studies are crucial to assess the impact of policies and practices during a pandemic, select which should be retained and scaled nationally, and inform future policy on crisis management and disease mitigation. Moreover, studies related to HCW need more attention considering the need to optimize health systems and trust in health facilities. Jakarta province, a megacity, the capital city and the COVID-19 hotspot in Indonesia, serves as a national and global model for developing urban health programs, and lessons learned regarding COVID-19 mitigation have value for other cities in Indonesia, and for LMIC and high-income countries.

This study sought to assess whether intervention policies for HCW were effective by comparing trends of COVID-19 cases and deaths among HCWs with non-HCWs during the initial phase of the pandemic in Jakarta province covering the first 8 months. We analysed the risk of COVID-19 cases and mortality in HCW compared to non-HCW in the context of policy formulation and implementation in reducing the transmission among HCWs during this critical period to gain insights for future outbreak management.

## Methods

### Study design and data collection

We used three sources of secondary data for the COVID-19 cases. First, from the Jakarta provincial government Executive Information Systems, we obtained individual-level data for COVID-19 suspected and confirmed cases from March to October 2020 on sociodemographic characteristics, symptoms, comorbidities and COVID-19 diagnosis history (i.e. confirmed cases and deaths). Second, from the Jakarta Smart City program we obtained individual-level case data which had been integrated with data from the Ministry of Health of the Republic of Indonesia. Third, we obtained data from referral laboratories that reported the COVID-19 test results to the provincial government. The data were merged for each individual in the respective databases, resulting in 1,230,071 COVID-19 and non-COVID-19 cases defined by results from SARS-CoV-2 polymerase chain reaction (PCR) assessment of nasopharyngeal swabs. These cases of COVID-19 included both inpatient and outpatient cases that were registered in Jakarta provincial government data.

We included only PCR-confirmed cases of COVID-19. We did not use cases reporting only symptoms and clinical presentation or antigen rapid tests (ART) because they were not adequate for diagnosis of COVID-19, especially in 2020. In some cases, ART had been used for screening before referral to a primary health centre (i.e. dental or maternal and child polyclinic) for PCR diagnosis. The results from ART were used to quantify persons mandated to self-isolate.

We categorized the patients into two groups, i.e. suspected and confirmed cases. Suspected cases were patients who had signs of acute respiratory infection or contact histories with confirmed cases of COVID-19. Meanwhile, confirmed cases were patients who had positive results of PCR test with or without symptoms [19].

The mortality data were also captured from several sources. The DKI Jakarta Public Health Office recorded daily mortality, and those data were cleaned and matched with mortality case data from the offices of cemeteries. This mortality rate data conformed to the required procedures for COVID-19 deaths reporting, with reports from the district health office surveillance website, and with data from the hospital information system. Redundant cases and deaths (i.e. COVID-19 for the same individual and the same day) and those with missing data for the key variables of interest were removed. Case fatality rate was calculated by dividing the number of death cases by number of confirmed cases. From these data, we selected adult subjects (aged ≥18 years) and categorized them into HCW and non-HCW based on their current occupation and location of work. We defined HCWs as those who worked in healthcare facilities, including persons with or without a medical degree. Government policies, including HCW-related policies, were obtained from the official website of the Jakarta provincial government (https://corona.jakarta.go.id/id/kebijakan), where written documentation of each policy was provided. Two authors (DR and KRE) independently extracted the data on the policies from the website, and discrepancies were discussed with the senior authors. We further classified HCW-related policies into hospital-based, community-based and well-being-related. The study protocol was approved by the Research Ethics Committee of the Faculty of Medicine, Universitas Indonesia, Dr. Cipto Mangunkusumo General Hospital (ethical approval number: 20030331). The Research Ethics Committee waived the requirement for written informed consent and approved the sharing of anonymized data based on the ethical issues, logistics and urgency of this work.

### Data analysis

Data were cleaned using Python and analysed using R Foundation for Statistical Computing (Vienna, Austria). First, we compared the baseline characteristics of HCWs and non-HCWs using Chi-square tests for two proportions. We identified the proportion of SARS-CoV-2 infections and COVID-19 deaths and compared them between HCW and non-HCW for each month from March to October 2020. To determine the effect of month on the odds ratio (OR) of COVID-19 cases and deaths, we constructed a generalized linear mixed-effect regression (GLMER) model with a binomial family and logit link function. We used a multilevel approach by inferring the probability of a HCW being tested for COVID-19 given the month (i.e. Swab P (HCW|Month), Death P (HCW|Month)). This approach was based on the differences in monthly trends in 2020 which may have been due to varying government policies. Hence, we integrated the timeline of government policies, including HCW-related policies, to understand the trend of SARS-CoV-2 infections and COVID-19 deaths in response to these policies. To enrich our understanding of the dynamics of COVID-19 cases and deaths among HCW, we included data on the number of patients isolated for COVID-19 and bed capacity in all referral hospitals. Statistical significance was set at *p* value <.05.

## Results

From the initial dataset of 1,230,071 suspected and confirmed COVID-19 cases in Jakarta province, we selected 894,487 adults (aged ≥18 years), consisting of 14,413 cases (5405 suspected; 9008 confirmed) among HCWs and 880,074 (501,586 suspected; 378,488 confirmed) cases among non-HCWs. The infection rate in HCW was 62.49%, and in non-HCW was 43.00%. Table 1 presents the baseline characteristics of HCWs and non-HCWs, most of whom were residents of Jakarta province. Similar proportions of subjects aged ≥40 and ≥65 years were found across suspected and confirmed cases between HCW and non-HCW. We found that contact history with COVID-19 patients in confirmed cases was higher in the HCW group (39.15%) than in the non-HCW group (29.37%). While COVID-19 symptoms varied, cough (23.41%), sore throat (13.43%) and fever (12.28%) were the most common symptoms. Only a small number of subjects had comorbidities, with hypertension (2.96%) being the most common. Our study found that the CFR was similar between HCW and non-HCW (2.32% vs. 2.40%, respectively).

**Table 1.** Baseline characteristics of suspected and confirmed COVID-19 cases among HCWs and non-HCWs in Jakarta province (*n* = 894,487).

Characteristic	HCW		Non-HCW	
	(*n* = 14,413)		(*n* = 880,074)	
	Suspected	Confirmed	Suspected	Confirmed
	(*n* = 5405)	(*n* = 9008)	(*n* = 501,586)	(*n* = 378,488)
	*n* (%)	*n* (%)	*n* (%)	*n* (%)
Aged ≥40 years old	2424 (44.85)	3930 (43.63)	205,040 (40.88)	177,697 (46.95)
Aged ≥65 years old	289 (5.35)	502 (5.57)	21,695 (4.33)	23,328 (6.16)
Sex (female)	3207 (59.33)	4552 (50.53)	229,536 (45.76)	179,212 (47.35)
Jakarta’s resident	5360 (99.17)	6507 (72.24)	500,620 (99.81)	314,471 (83.09)
Contact history	107 (1.98)	3527 (39.15)	820 (0.16)	111,180 (29.37)
Symptoms				
Cough	52 (0.96)	2109 (23.41)	1229 (0.25)	52,264 (13.81)
Fever	40 (0.74)	1106 (12.28)	1020 (0.20)	22,268 (5.88)
Diarrhoea	5 (0.09)	305 (3.39)	138 (0.03)	5691 (1.50)
Malaise	19 (0.35)	870 (9.66)	516 (0.10)	16,164 (4.27)
Myalgia	21 (0.39)	748 (8.30)	302 (0.06)	13,124 (3.47)
Nausea	12 (0.22)	553 (6.14)	398 (0.08)	10,505 (2.78)
Difficult breath	11 (0.20)	740 (8.21)	666 (2.82)	22,958 (6.07)
Headache	29 (0.54)	974 (10.81)	406 (0.08)	21,295 (5.63)
Sore throat	39 (0.72)	1210 (13.43)	619 (0.12)	26,017 (6.87)
Abdominal pain	7 (0.13)	322 (3.57)	116 (0.02)	7494 (1.98)
Comorbidities				
Hypertension	4 (0.07)	267 (2.96)	252 (0.05)	5749 (1.52)
Diabetes	1 (0.02)	123 (1.37)	175 (0.03)	3315 (0.88)
COPD	1 (0.02)	68 (0.75)	45 (0.01)	851 (0.22)
Liver	1 (0.02)	6 (0.07)	9 (0.001)	273 (0.07)
Asthma	0	4 (0.04)	0	178 (0.05)
Death status				
Non-death	5402 (99.94)	8799 (97.68)	501,151 (99.91)	369,386 (97.60)
Death	3 (0.06)	209 (2.32)	435 (0.09)	9102 (2.40)
CFR, %	–	2.32	–	2.40

CFR: case fatality rate; COPD: chronic obstructive pulmonary disease.

Only liver comorbidity and asthma did not differ significantly.

**Figure 1.**
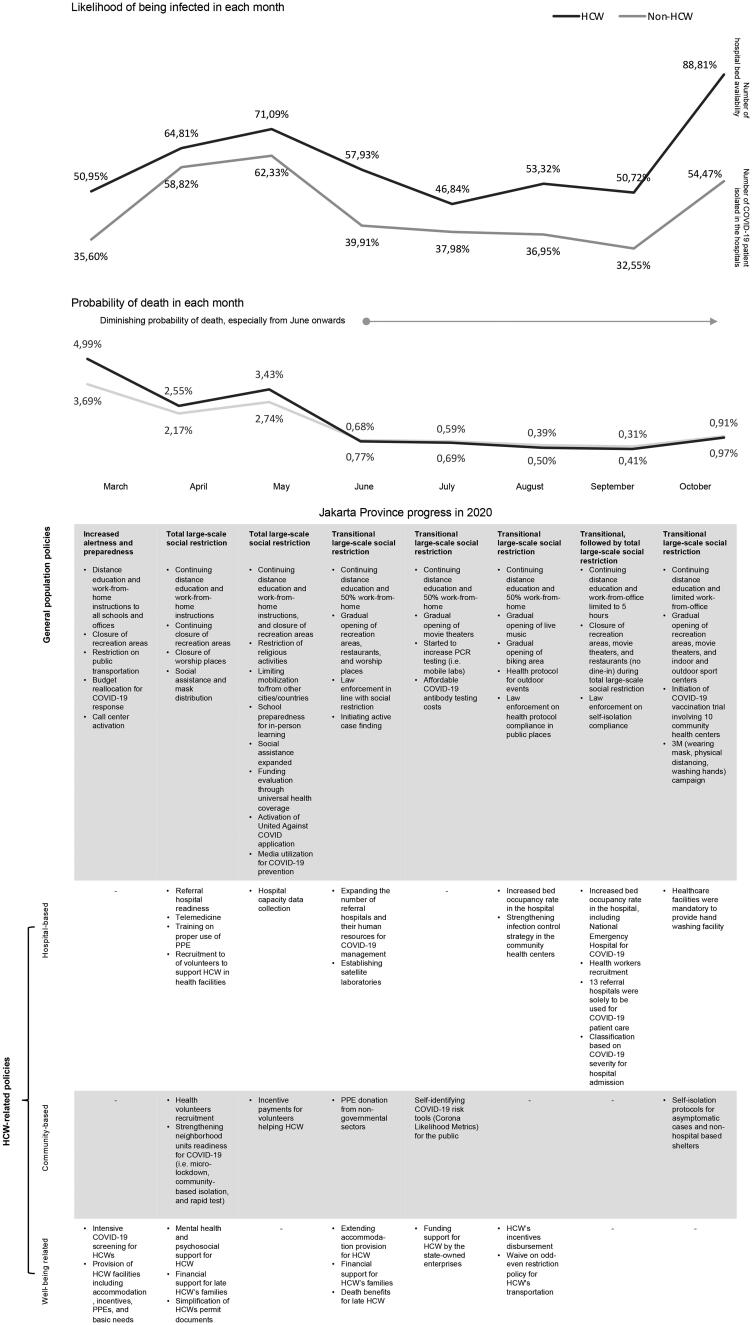
Likelihood of COVID-19 cases and deaths among HCWs and non-HCWs in Jakarta, and the number of hospitals’ beds and patients, in response to the dynamics of government policies.

**Figure 2.**
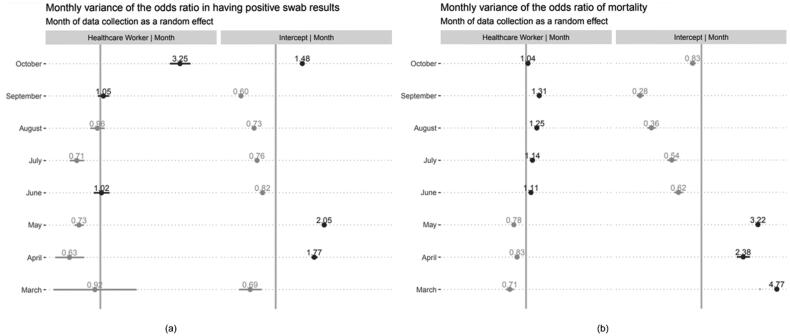
Comparison of monthly variance of the odds ratio between HCW and non-HCW (intercept) in having COVID-19 (a) confirmed cases and (b) mortality.

Figure 1 depicts the likelihood of confirmed COVID-19 cases and deaths from March to October 2020 in response to the dynamics of government policies consisting of general population and HCW-related policies. GLMER analyses demonstrated that the OR of COVID-19 confirmed cases for HCW was 2.04 (95% CI 2.00–2.08; *p* < .001) compared to non-HCW. Likelihood comparisons across months consistently showed that HCW had a higher probability of being infected with SARS-CoV-2. However, Figure 2 reveals that from April to July 2020, except for June, ORs <1 were observed for HCW, whereas ORs >1 were observed from August to October 2020. This trend aligns with the trend in the number of COVID-19 patients in hospitals that surged from August to October 2020. For COVID-19 deaths, the trend showed a diminishing probability of death in both groups, especially from June onwards. Based on the GLMER results, no significant differences were found in the likelihood of death among HCWs compared to non-HCWs (OR 1.02, 95% CI 0.93–1.11; *p* = .65).

### Government general population and HCW-related policies during the initial period of the pandemic

We identified the trends of the ORs for COVID-19 confirmed cases, which described lower odds from April to July 2020 (i.e. April-to-July phase), followed by a gradual increase in the odds from August to October 2020 (i.e. August-to-October phase) among HCWs compared to non-HCWs. The gradual increase in the August to October phase paralleled the surge in COVID-19 patients in hospitals. Based on these findings, we compared the government policies implemented across months following these trends during the initial 8-month period of the pandemic.

The April-to-July 2020 phase started with the implementation of the total large-scale social restriction (LSSR) as part of the general policies in early April, in response to the first confirmed COVID-19 case on 2 March 2020. In March, the government increased the alertness and preparedness of the general public towards COVID-19 transmission risk. Distance education and work-from-home (WFH) were ordered for all schools and offices, recreation areas were closed, and public transportation was restricted. The local government budget was reallocated for the COVID-19 response. The Jakarta provincial government also activated a call centre for public responses. In addition, the government ordered the closure of places of worship and initiated social assistance and mask distribution. The total LSSR continued until May 2020, together with the restriction of religious activities since the month coincided with Eid Al-Fitr, a Muslim holiday, at the end of the fasting month. One policy related to religious restrictions was the prohibition of mass travel, in addition to limiting mobilization to or from other cities that traditionally accompanied Eid Al-Fitr. Social assistance was expanded and funding from universal health coverage was allocated to strengthen funding for the COVID-19 response. The United Against COVID smart phone application was launched by the national government and media to promote COVID-19 prevention. As preparation to ease the LSSR, school preparedness for in-person learning was initiated. From June to July, the government implemented a transitional LSSR, with fewer restrictions. Public places and offices were gradually opened at half-capacity, with an enforced protocol for COVID-19 prevention. In accordance with the transitional phase, the government initiated active case finding, set prices for affordable PCR testing, and accepted the use of COVID-19 antigen and antibody tests for diagnosis. Moreover, law enforcement was ordered to maintain social restrictions retained in the transitional LSSR.

Importantly, in the April-to-July 2020 phase, the government implemented some HCW-related policies that combined hospital-based, community-based and well-being-related aspects. Hospital-based policies were initiated and improved throughout the phase, including improving referral hospital readiness, activating telemedicine, PPE training and hospital-based data collection. Importantly, these efforts were supported by community-based initiatives, including health volunteer recruitment with incentives, community-based self-isolation programs and self-identifying COVID-19 risk for the public. HCW well-being-related policies were initiated, including psychosocial support, extended provision of accommodations to prevent exposure to their families, and support for HCW’s families, i.e. providing permission for HCW’s children to register to public schools with no tests. Altogether, the HCW-related policies in the April-to-July 2020 phase helped reduce the patient load at hospitals during a surge in cases that occurred during the same period (Figure 1).

However, during the August-to-October 2020 phase, the transitional LSSR was implemented throughout, except in the last two weeks of September 2020. Importantly, this phase was marked by a gradual opening of recreational activities (i.e. live music, biking areas, movie theatres and sports centres) but was not followed by the expansion of community-based and well-being-related policies to provide more support to HCW. In contrast, we observed an increasing trend in the number of COVID-19 patients in hospitals, with a peak in October. Taken together, the August-to-October 2020 phase was characterized by hospital overcapacity with inadequate expansion of community-based and HCW well-being-related policies, which may have led to an increased risk of SARS-CoV-2 infection among HCWs.

## Discussion

In this study, we found a 104% increase in the odds of testing positive for COVID-19 in HCW compared with non-HCW. Despite the higher OR for COVID-19 infection, the CFR and odds of death were not significantly different between HCW and non-HCW. Importantly, during the 8-month initial period of the pandemic, the OR of COVID-19 infection among HCWs decreased in months when particular HCW-related policies were implemented (i.e. April to July 2020), which emphasized the improvement of hospital readiness, supporting the well-being of the HCW, and activation of public participation through community-based self-isolation and risk assessment. In contrast, the OR gradually increased among HCWs when the social restriction loosened, and in turn, the Jakarta provincial government documented an increased number of patients isolated in health facilities for COVID-19 (i.e. August-to-October 2020 phase). Interestingly, this trend was also observed in the number of patients isolated in hospitals, showing an increased rate during the same period.

This study is among the largest to evaluate SARS-CoV-2 infections and deaths in Jakarta, the capital city and one of the COVID-19 hotspots in Indonesia. This is also among the first studies to compare COVID-19 outcomes between HCW and non-HCW in an LMIC. We included all cases reported by the government surveillance system in the city, thus reducing selection bias. The Jakarta provincial government accelerated the development of information and database systems for COVID-19 reporting during the first months of the pandemic, although the Ministry of Health of the Republic of Indonesia had yet to establish such systems. Additionally, HCWs and non-HCWs had comparable characteristics (i.e. age, sex and comorbidities), except for contact history, which was higher in HCWs (39.15%) than in non-HCWs (29.37%). We noted the low incidence of comorbidities (hypertension, diabetes mellitus, chronic obstructive pulmonary disease and liver disease) in both HCW and non-HCW. The data on comorbidities must be manually entered into a system in order to be captured by the central health office. However, due to a greater workload during the first wave of the pandemic, the hospitals could not appropriately record all comorbidities of the patients. Therefore, the low comorbidity rates might not represent the real condition in the community.

Our findings demonstrated a higher prevalence of COVID-19 cases among HCWs than in other studies in the United States (US) (2.7–4.5%), Iran (5.62%) and China (1.1–11.9%) [20–24]. This could be due, in part, to the inclusion of only suspected cases of COVID-19 (i.e. symptomatic subjects and those with a contact history). However, our results on increased odds of infection (OR 2.04) among HCWs were similar to the nationwide study conducted in the US (HR 2.80) and the United Kingdom (UK) (HR 12.52) among HCWs.

Our study observed similar trends in CFR between HCW and non-HCW. In March 2020, HCWs were one of the national priority groups for access to COVID-19 testing, thus enabling early treatment for confirmed cases among HCWs [25]. To the best of our knowledge, information on mortality trends in two distinct groups of HCWs and non-HCWs from previous studies is lacking. A study in the US showed lower mortality rates among HCWs (1%) than among non-HCWs (8%) [26], while another study in the UK demonstrated a higher risk of severe COVID-19 in essential workers, including HCWs [27].

HCWs have consistently been associated with higher odds of SARS-CoV-2 infection. In our study, the monthly odds of COVID-19 cases among HCWs were consistently higher than those of non-HCWs. Various factors may account for these findings, such as inadequate personal protective equipment (PPE) provision and use, higher exposure to COVID-19 cases, and prevention measures in activities outside the work shift. PPE shortage in most of the healthcare facilities in Indonesia, including in Jakarta, in the first few months of the pandemic has been documented and was due to the high demand coming not only from medical professionals but also from the public [10]. This condition would lead to inadequate PPE use and result in PPE reuse, which has been associated with a higher risk for COVID-19 in a previous study, and could affect the exposure risk of HCW [20]. Moreover, higher exposure to COVID-19, i.e. caring for patients with suspected or documented COVID-19, also increased the risk of COVID-19 infection among HCWs, even when they reported adequate PPE use. This aligns with our findings, in which HCW showed a higher COVID-19 contact history compared to non-HCW, although data on PPE use were absent in this study. This may suggest the importance of re-evaluating the PPE level recommendations and, more importantly, the correct application and adherence to the recommendations. Lastly, alongside the implementation of PPE use during the work shift, it is worth mentioning that reinforcement of preventive measures is needed outside the clinical situation, including breaks and meal times.

The Jakarta provincial government implemented several HCW-related policies that were adaptable to the dynamics of COVID-19 cases and financially supported them through budget reallocations from the local government. Our study indicates that community-based and well-being-related policies as part of COVID-19 prevention and management protocols are among the key factors that may reduce the risk of COVID-19 among HCWs. Various countries have explored community-based approaches to cope with the pandemic [28,29]. Moreover, a few countries have also included public participation in the decision-making process of COVID-19 policies [30,31]. Community resource utilization during the COVID-19 pandemic is vital and feasible under restricted circumstances to help decrease the burden and load in hospitals [32]. Its implementation in Jakarta province was accompanied by improving hospital readiness, including enhancing medical waste protocols, increasing human resources, monitoring the bed occupancy rate, and promoting the well-being of HCW through accommodation provision, family and mental support during the time of increased loads in healthcare facilities. Mental support for HCW must not be overlooked, as previous studies have highlighted psychological exhaustion and a range of mental health impacts among HCWs, including fear of contracting COVID-19 and infecting loved ones, fear of early death, obligation to work during the pandemic due to financial matters, and being in a psychologically conflicted position between lack of PPE, professionalism and moral responsibility of caring for patients [33–35]. Collectively, combined efforts to address these issues might prevent hospital overcapacities, reduce workload and contact among HCWs during a surge of COVID-19 cases, while still ensuring the well-being of HCW, resulting in decreased odds of COVID-19 cases. Interestingly, during the August-to-October 2020 phase marked with loosened social restrictions, the scale-up of such combined efforts was not observed, resulting in inadequate support of hospital-based policies in preventing hospital overload and COVID-19 contact and risk among HCWs.

There were possibilities that other factors besides changes in policy could affect the outcomes in COVID-19. We noted an increase in the ICU bed occupancy rate to 71% in August 2020, which continued to increase through September 2020 [36]. There was also limited access to therapeutics during that time. The National Agency for Drug and Food Control of Indonesia released the Emergency Use Authorization (EUA) for Favipiravir on 3 September 2020, while the EUA for Remdesivir was released on 19 September 2020 [37]. However, these therapeutic drugs were expensive and evidence of their efficacy was limited. We could not confirm whether there was a shortage of ventilators or equipment during that time. All of these may have contributed to the sharp increase in the likelihood of being infected, both among HCWs and non-HCWs, that was observed in October 2020, despite the implementation of the total LSSR in mid-September 2020. Often, changes in policy occur in response to the current situation, e.g. the total LSSR in mid-September 2020 was decided due to hospital overcapacity.

While our study identified important findings to ensure collective support from a combined effort for HCW during the unprecedented situation of the pandemic, a few limitations should be taken into consideration. First, detailed information on PPE, such as PPE availability and practice of PPE reuse, as an important determinant that may increase risk, was not evaluated in this study. Hence, for future studies, we suggest expanded data collection on HCW’s characteristics, including their place of work (i.e. primary vs. secondary vs. tertiary health care) and adherence to the PPE recommendations, including during the non-work shift time. Second, concerning the community-based approach of COVID-19 management, compliance with the protocols for this approach warrants more intensive monitoring. Previous studies have also identified challenges to non-adherence in this approach [38,39].

Our study is consistent with a previous study showing the importance of supporting and ensuring the well-being of HCW, as they consistently face higher COVID-19 risks and may contribute to the spread of the infection in the community. Our findings add that collective support through combined efforts in HCW-related policies with community-based and well-being-related approaches, alongside hospital-based policies, is crucial to prevent hospital overcapacity, especially during the initial period of the pandemic. This would help reduce COVID-19 contact and workload, while promoting pandemic resilience among HCWs. Another lesson learned for future pandemics is to enhance surveillance while any vaccine development is in progress. During the pandemic period herein, there had not been any SARS-CoV-2 vaccines developed and provided to HCW. The first vaccine (Sinovac Biotech’s CoronaVac) was available in Indonesia on 13 January 2021. Therefore, there is no impact of vaccination on the outcomes of this study. It is worth mentioning that the results of the comparison between HCW and non-HCW may depend on the settings and measures taken by the local government concerning COVID-19 prevention. The variation in results across regions or settings warrants further investigation.

## Conclusions

In summary, our analysis found that, while government policies appeared to lower cases both in hospitals and in the community, HCW had a higher risk of SARS-COV-2 infection but a similar risk of death from COVID-19 as non-HCW. Although many factors could have contributed to these findings, our study found that government policies aimed at reducing hospital loads and promoting HCW’s well-being helped to reduce the spread of SARS-COV-2 infection among HCWs, particularly in the early stages of the pandemic as a crucial time in crisis management. Future studies are warranted with expanded sociodemographic, PPEadherence, and vaccine coverage data among HCWs, and studies should be conducted in other regions with different local government policies.

## Data Availability

Data are available on reasonable request.
