# Correction

**DOI:** 10.1080/07853890.2024.2317590

**Published:** 2024-02-16

**Article title:** The impact of government pandemic policies on the vulnerability of healthcare workers to SARS-CoV-2 infection and mortality in Jakarta Province, Indonesia

**Authors**: Agustina, R., Rianda, D., Lamuri, A., Ekawidyani, K. R., Siregar, D. A. S., Sari, D. S., Wulan, P. M., Devana, N. D., Syam, A. F., Rahyussalim, A. J., Handayani, D.O., Widyastuti, W., Shankar, A. H., & Salama, N.

**Journal**: *Annals of Medicine*

**Bibliometrics:** Volume 55, Number 2, pages 2293306

**DOI**: https://doi.org/10.1080/07853890.2023.2293306

When this article was first published online, the gray shades in the portion depicting “Number of Hospital Bed Availability’ and “Number of COVID-19 Patient Isolated in the Hospital” were missing in Figure 1.

The Figure has now been updated and is displayed on the following page.

**Figure 1.**
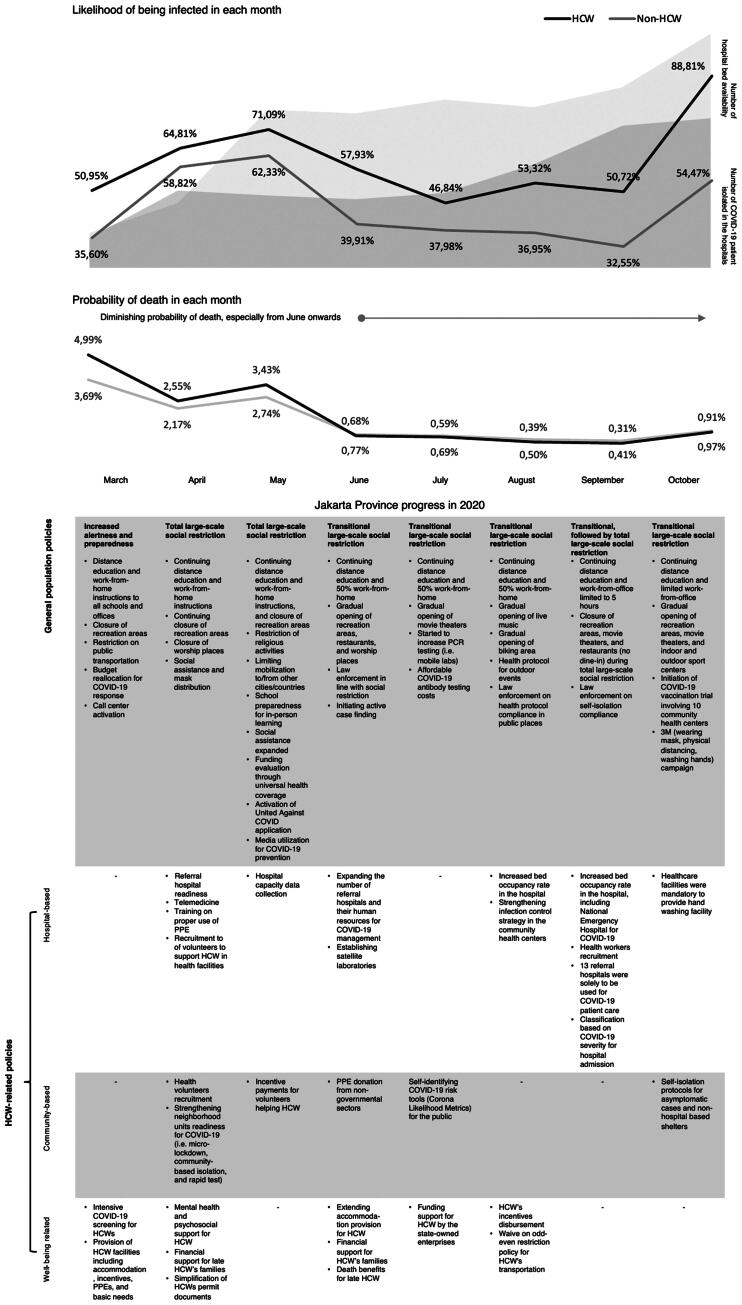
Likelihood of COVID-19 cases and deaths among HCWs and non-HCWs in Jakarta, and the number of hospitals’ beds and patients, in response to the dynamics of government policies.

